# Divergent Evolution of TRC Genes in Mammalian Niche Adaptation

**DOI:** 10.3389/fimmu.2019.00871

**Published:** 2019-04-24

**Authors:** Zepeng Zhang, Yuan Mu, Lei Shan, Di Sun, Weijian Guo, Zhenpeng Yu, Ran Tian, Shixia Xu, Guang Yang

**Affiliations:** Jiangsu Key Laboratory for Biodiversity and Biotechnology, College of Life Sciences, Nanjing Normal University, Nanjing, China

**Keywords:** TRC genes, mammals, correlation, divergent evolution, niches

## Abstract

Mammals inhabit a wide variety of ecological niches, which in turn can be affected by various ecological factors, especially in relation to immunity. The canonical TRC repertoire (*TRAC, TRBC, TRGC*, and *TRDC*) codes C regions of T cell receptor chains that form the primary antigen receptors involved in the activation of cellular immunity. At present, little is known about the correlation between the evolution of mammalian TRC genes and ecological factors. In this study, four types canonical of TRC genes were identified from 37 mammalian species. Phylogenetic comparative methods (phyANOVA and PGLS) and selective pressure analyses among different groups of ecological factors (habitat, diet, and sociality) were carried out. The results showed that habitat was the major ecological factor shaping mammalian TRC repertoires. Specifically, trade-off between *TRGC* numbers and positive selection of *TRAC* and the balanced evolutionary rates between *TRAC* and *TRDC* genes were speculated as two main mechanisms in adaption to habitat and sociality. Overall, our study suggested divergent mechanisms for the evolution of TRCs, prompting mammalian immunity adaptions within diverse niches.

## Introduction

Mammals have successfully colonized the earth owing to their inhabiting of a wide range of ecological niches with diverse habitats, diets and social structures (1). With changes in habitats, diets, and social groups (e.g., different group size), the pathogens encountered by animals would be changed, thus, new variations in genes referred to immunity would be maintained.

Studies on the relationships between ecological factors and variations of immune genes have emerged in recent years. Recently, molecular adaptations in pattern recognition receptors (PRRs) were highlighted among mammals with diverse niches (habitat, diet, and living pattern) (2). Cetaceans are good models to assess the evolutionary changes of habitat transitions from land to water. Strong evidence of positive selection for the Toll-like receptor four (*TLR4*) gene was identified in the periods of evolutionary transition from land to semi-aquatic and from semi-aquatic to full aquatic, suggesting adaptive evolution of cetacean *TLR4* during habitat transitions (3). Studies have also been conducted in bats, which, as the reservoirs of a number of emerging viruses, have received much attention in the field of immunity (4). By comparing the genome of *Myotis davidii* (insectivorous and living in rock cavities) and *Pteropus alecto* (frugivorous or nectarivorous and living in trees, mangroves and rainforest), Zhang et al. (5) reported that considerable gene duplication of some members of the leukocyte receptor complex (LRC) genes were identified in *M. davidii* but not in *P. alecto*, suggesting diverse adaptions between these two niche-distinct bats in innate immunity. However, these studies have concentrated on some representative species and the innate immunity, which is relatively more conserved than adaptive immunity, which is sensitive to the environment.

T cell receptors (TCRs) are the primary antigen receptors involved in the activation of cellular immunity which recognize the processed protein antigens presented by major histocompatibility complex (MHC) (6). There are two canonical types of TCRs present on the surface of T cells: αβ TCRs and γδ TCRs. The former is composed of a heterodimer formed by α and β chains, and the latter is characterized by a heterodimer of γ and δ chains (7). The TRC repertoire is the set of immunogloblin superfamily (IgSF) genes, mainly including *TRAC, TRBC, TRGC*, and *TRDC*, which code the constant (C) regions: a constant (C) domain, a connecting region (CO), a transmembrane region (TM), and a cytoplasmic region (CY) of corresponding TCR chains. The C region provides the scaffolding for TCR molecules and the mechanism for signal transduction into the cell (8). The well-annotated TRC genes of human (*Homo sapiens*) can be referred to as an example, each of the four canonical types of genes consist of four exons, except for *TRGC* gene which has only three. Only the first three exons are translatable except the EX4 exon of *TRBC1* and *TRBC2* which encode the short intracytoplasmic region (7). Recently, an atypical TCR chain named TCRμ was identified in monotremes and marsupials, which was encoded by TRM locus (9–11). This unconventional TCR chain in monotremes and marsupials is expressed in a form containing double V domains, which is different from the conventional TCR in other mammals (10).

Previous research on TRC genes was mainly focused on sequence characteristics of single types of TRCs by means of gene cloning and molecular hybridization (12–14). Variation in sequence length and amino acid residues on *TRGC* genes were reported in some species, including human, dog (*Canis lupus familiaris*), platypus (*Ornithorhynchus anatinus*), rabbit (*Oryctolagus cuniculus*), and ruminants (14–18). Recent whole-genome sequencing of different mammals has made it possible to retrieve entire gene sequences from their genomes. Generally, the TRC genes have shown low diversity in copy number and sequence variation in the examined genomes of cow (*Bos taurus)*, sheep (*Ovis aries)*, and bottlenose dolphin (*Tursiops truncatus*) (19–21). However, little is known about the correlation between ecological factors and the evolution of mammalian TRC genes as adaptive immunity sensors. Is there a correlation between the evolution of TRC repertoires and ecological factors? What is the evolutionary pattern of mammalian TRCs in niche adaptions? To explore these issues, four canonical types of TRC genes (*TRAC, TRBC, TRGC*, and *TRDC*) were retrieved from 37 species across eight mammalian taxa that possess different niches, i.e., habitat (aquatic, semi-aquatic, terrestrial, and aerial), diet (carnivore, frugivore, insectivore, herbivore, and omnivore), and sociability (social and solitary) and subjected to comparative phylogenetic and molecular evolutionary analysis to investigate the influence of these genes on niche adaptation and their association with ecological variations.

## Materials and Methods

### Taxon Coverage and TRC Identification

We downloaded the available TRCs of five species, human, mouse (*Mus musculus*), dog, cow and sheep, from IMGT®–the international ImMunoGeneTics information system® http://www.imgt.org. The sequences are listed in Table S1. Another 32 mammal genomes were also scanned using BLASTN to retrieve the TRC genes (22). The exon/intron boundaries of each gene were referenced by human TRCs in IMGT®. The detailed genome information has been compiled in Table 1. The identified TRC genes retrieved from genomes were further checked by blast searches against the non-redundant database from GenBank. Gene trees reconstructed using IQ-TREE (23) and MrBayes (24) were employed to further classify each gene to its corresponding cluster. The newly identified TRC genes were categorized into intact genes (continuous from the start to stop codon and putative to be functional), partial genes (only part of the gene or single exons), and pseudogenes (ORF is disrupted by frameshift mutations and/or unexpected stop codons) based on the amino acid alignment and blast results. The newly identified gene sequences from genomes are given in Table S1 and the scaffold information was listed in Table S2.

**Table 1:** Genomic information of 32 mammal genomes used in this study.

**Classification**	**Species name**	**Assembly**	**Date**	**Coverage and sequence technology**	**Contig N50 (× 10^**3**^)**
Cetartiodactyla	*Tursiops truncates* (Ttru)	NIST Tur_tru v1	Dec.2016	114.5x Illumina	44.3
	*Orcinus orca* (Oorc)	Oorc_1.1	Jan.2013	200x Illumina	70.3
	*Neophocaena asiaeorientalis* (Neas)	Neophocaena_asiaeorientalis_V1	Apr.2018	106x Illumina	86
	*Delphinapterus leucas* (Dleu)	ASM228892v2	Nov.2017	117.0x Illumina	159.1
	*Lipotes vexillifer* (Lvex)	Lipotes_vexillifer_v1	Jul.2013	115x Illumina	31.9
	*Physeter macrocephalus* (Pmac)	Physeter_macrocephalus-2.0.2	Sep.2013	75x Illumina	35.3
	*Balaenoptera acutorostrata* (Bacu)	BalAcu1.0	Oct.2013	92x Illumina	22.7
	*Balaena mysticetus* (Bmys)	1	Jan.2015	150x Illumina	34.8
	*Sus scrofa* (Sscr)	Sscrofa11.1	Feb.2017	65.0x PacBio	48231.3
	*Hippopotamus amphibious* (Hamp)	ASM299558v1	Mar.2018	55x Illumina	34
Carnivora	*Ursus maritimus* (Umar)	UrsMar_1.0	May.2014	101x Illumina	46.5
	*Odobenus rosmarus* (Oros)	Oros_1.0	Jan.2013	200x Illumina	90
	*Leptonychotes weddellii* (Lwed)	LepWed1.0	Mar.2013	82x Illumina	23.7
	*Ailuropoda melanoleuca* (Amel)	AilMel_1.0	Dec.2009	60x Illumina	39.9
Chiroptera	*Rhinolophus sinicus (*Rsin)	ASM188883v1	Dec.2016	146.44x Illumina	37.8
	*R. ferrumequinum* (Rfer)	ASM46549v1	Sep.2013	17.0x Illumina	11.7
	*Pteronotus parnellii* (Ppar)	ASM46540v1	Sep.2013	17.0x Illumina	9.5
	*Myotis lucifugus* (Mluc)	Myoluc2.0	Sep.2010	7x Sanger	64.3
	*M. davidii* (Mdav)	ASM32734v1	Dec.2012	110x Illumina	15.2
	*M. brandtii* (Mbra)	ASM41265v1	Jun.2013	120x Illumina	23.3
	*Miniopterus natalensis* (Mnat)	Mnat.v1	Mar.2016	77.0x Illumina	29.8
	*Megaderma lyra* (Mlyr)	ASM46534v1	Sep.2013	18.0x Illumina	7
	*Hipposideros armiger* (Harm)	ASM189008v1	Dec.2016	218.6x Illumina	39.9
	*Eptesicus fuscus* (Efus)	EptFus1.0	Nov.2012	84x Illumina	21.4
	*Eidolon helvum* (Ehel)	ASM46528v1	Sep.2013	18.0x Illumina	12.7
	*Rousettus aegyptiacus* (Raeg)	Raegyp2.0	Mar.2016	169.2x Illumina; PacBio	1489
	*Pteropus vampyrus* (Pvam)	Pvam_2.0	Dec.2014	188.0x Illumina	21.9
	*P. alecto* (Pale)	ASM32557v1	Dec.2013	110x Illumina	31.8
Euarchontoglires	*Nomascus leucogenys* (Nleu)	Nleu_3.0	Dec.2012	5.6x Sanger	35.1
Afrotheria	*Trichechus manatus* (Tman)	TriManLat1.0	Jan.2012	150x Illumina	37.8
	*Loxodonta africana* (Lafr)	Loxafr3.0	Jul.2009	7x Sanger, ABI	69
Monotremata	*Ornithorhynchus anatinus* (Oana)	ASM296699v1^a^	Mar.2018	90x PacBio;Illumina	7578.7
	*Ornithorhynchus anatinus* (Oana)	Ornithorhynchus_anatinus-5.0.1^b^	Jun. 2011	7x shotgun	11.6

*Abbreviation of the species is listed in parentheses*.

a *This genome was used to identify TRAC, TRBC, and TRGC sequences*.

b *This version of genome of platypus was used to identify TRDC and TRMC sequences for comparison to the results from previous studies*.

### Gene Tree Reconstruction

The dataset, incorporating all of the 281 identified sequences, was aligned using MEGA 6 (25). Gene trees were reconstructed using maximum likelihood (ML) method in IQtree 1.6.5 (23) and Bayesian inference (BI) in MrBayes 3.2.2 (24). The best-fit model for the ML tree was chosen as suggested by the IQ-TREE model test tool with Bayesian Information Criterion. To assess branch support, ultrafast bootstrap approximation (UFboot) was employed with 1,000 replicates (26) as well as the SH-like approximate likelihood ratio test (SH-aLRT), also with 1,000 bootstrap replicates (27). For the BI tree, the best-fit model was chosen using MrModeltest 2.3 (28) with the Akaike Information Criterion (AIC) (29). Bayesian inference analysis was run for 10^6^ generations with one cold and three heated Markov chains. The first 25% of the trees were discarded as burn-in, after examination of the output in Tracer 1.7 (30) to ensure a minimum estimated sample size (ESS) higher than 200 for all sample parameters.

### Phylogenetic Comparative Methods

We analyzed the evolution of TRC repertoires relative to niche adaptations using phylogenetic analysis of variance (phyANOVA). First, we categorized the 37 species according to the following niches: Categorical variables incorporated into this study were handled as follows, for habitat; aquatic (1), aerial (2), semiaquatic (3), and terrestrial (4), for diet; carnivore (1), frugivore (2), herbivore (3), insectivore (4), and omnivore (5), and for sociality; sociality (1) and solitary (2) (Table S3, Figure S1). Then we calculated the total gene number of TRCs in each species and these number were log transformed to meet the assumptions of normality of phyANOVA analyses. Tree file with divergence time was taken from TimeTree (http://www.timetree.org/) (31) and corrected according to the well-supported species phylogeny of Ranwez et al. (32), Teeling et al. (33), and Zhou et al. (34). Finally, phyANOVA analysis was performed using the R packages “ape” (35), “geiger” (36), and “phytool” (37). Moreover, we used phylogenetic generalized least squares (PGLS) regression (38) to investigate the relationship between the number of TRC genes and ecological factors while statistically controlling for phylogeny. PGLS regression analyses were performed using R with the caper packages (39).

### Molecular Evolutionary Analyses

Selective pressure was tested only on *TRAC* and *TRDC* genes because of the multiple-copy characteristics of *TRBC* and *TRGC* genes. Because the platypus *TRDC* shared low identity with other mammalian *TRDC*s, we removed this sequence from our dataset. To estimate the strength and form of selection acting on TRC genes, the alignment, along with the species trees modified from Ranwez et al. (32), Teeling et al. (33), and Zhou et al. (34) were analyzed with the CODEML program of PAML4 (40), which could estimate the rate of non-synonymous substitutions (*d*_N_) and the rate of synonymous substitutions (*d*_S_) among sites and branches. The ω ratio (*d*_N_ / *d*_S_) < 1, = 1, and >1 indicates purifying selection, neutral evolution and positive selection, respectively. The likelihood ratio test (LRT) with a χ^2^ distribution was used to determine the statistically significantly models compared with the null models at a threshold of *p* ≤ 0.05. Bayes empirical Bayes (BEB) analysis was used to identify sites under positive selection with posterior probabilities ≥ 0.95 (41).

First, random sites models, i.e., M8 (positive selection) vs. M8a (nearly neutral) (40) were applied to detect positively selected sites of TRCs in the 37 species. Then, three ML models, i.e., random-effect likelihood (REL), fixed-effect likelihood (FEL), and Fast, Unconstrained Bayesian AppRoximation (FUBAR) implemented on the Datamonkey webserver were used to detected divergent selection and positive selection (42, 43), with default settings and significance levels of 50, 0.1, and 0.9, respectively.

To detect branch specific evolutionary rates, we utilized the branch model (free-ratio vs. one-ratio model) in CODEML program to estimate ω ratio of each branch. To further compare the evolutionary rates of TRCs in response to discrepant niches of mammals, we finally applied Clade Model C (CmC), which permits a class of codon sites to evolve differently along the phylogeny (44). We partitioned the 37 species according to each of the niches displayed in Figure S1: habitat (aquatic, semi-aquatic, terrestrial, and aerial), diet (carnivorous, frugivorous, insectivorous, herbivorous, and omnivorous), and sociality (sociality and solitary). The best fitting model allowing for divergent evolutionary rates (ω_d_) and occurring among each partition of niche was compared with the null model M2a_rel (45), which does not permit divergence in the foreground clade but allows for an unconstrained ω.

## Results

### TRC Identification and Genetic Characteristics

Before identification of the four canonical TRCs, we first investigated *TRMC* from the genomes of platypus (Ornithorhynchus_anatinus-5.0.1) and opossum (MonDom5) taking the eight *TRMC* sequences of opossum (10) as queries. A total of 15 and 9 *TRMC* sequences were successfully retrieved from the genomes of platypus and opossum, respectively. Both of these numbers were higher than that from the identifications of Wang et al. (11) and Parra et al. (10). However, the gene number of *TRMC* (15) in platypus was consistent with that in Warren et al. (46). Sequence alignment for the newly identified 15 *TRMC*s in platypus and the *TRMC*s from previous studies (11) showed that these sequences shared overall mean 74% nucleotide identity (Figure S2). For opossum, although an accessorial *TRMC* was identified compared to Parra et al. (10), all these sequence shared overall mean 75.3% nucleotide identity (Figure S2), which hinted the same locus for these sequences. Phylogenetic analyses with BI and ML methods were further employed to verify these *TRMC*s from platypus and opossum. Notably, the platypus *TRMC* and opossum *TRMC* clustered with each other with 100% posterior probability, suggesting a close relationship between them. In addition, both trees revealed that the platypus and opossum *TRMC*s formed a well-supported monophyletic clade, which clustered with mammalian *TRDC*s (Figures S3,S4). This result was consistent with that from Wang et al. (11) where a close relationship between *TRMC* and *TRDC* genes was revealed.

To verify the hypothesis that TRM locus is only present in monotremes and marsupials, we further checked the genomes of African savanna elephant (*Loxodonta Africana*) and Florida manatee (*Trichechus manatus latirostris*), which located at the basal position of mammalian phylogeny, while no *TRMC*-like sequences were identified from these two genomes. Then three more genomes: human (*Homo sapiens*), Egyptian fruit bat (*Rousettus aegyptiacus*), and beluga (*Delphinapterus leucas*) were checked and no *TRMC*-like sequences were identified. Combined with the other eight mammals investigated in Parra et al. (10) where no *TRMC* were identified, we supported the ancient presence of TRM locus in mammals. Because of the uniqueness of *TRMC* in monotremes and marsupials, they didn't be included in further analysis.

**Figure 1:**
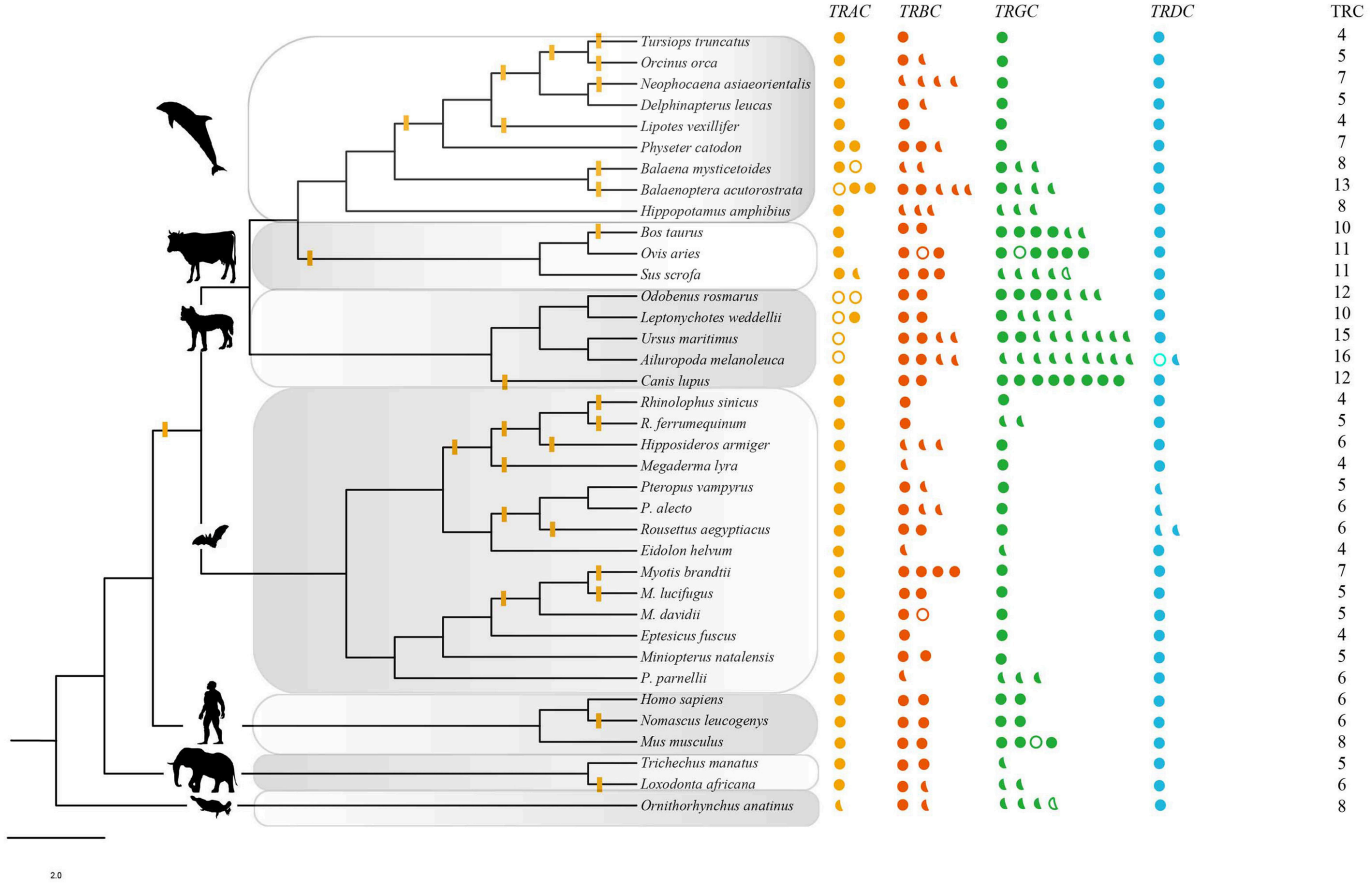
TRC repertoires in 37 species. Solid circles: intact genes; solid semicircles: partial segments; hollow circles and (or) semicircles: pseudogenes. The phylogenetic tree was modified from a widely accepted phylogeny of mammals (32–34). The branches with yellow bars indicated the evidence of positive selection estimated by free ratio. The intensity of positive signal in each taxon was calculated following the equation: intensity = total signal/total number of examined branches and nodes. Artiodactyla: 2/5, carnivora: 2/9, euarchontoglires: 1/5, afrotheria: 1/3. Yellow: *TRAC*, red: *TRBC*, green; *TRGC*, blue: *TRDC*.

For the four conventional TRC loci, a total of 269 sequences from 37 mammalian species were identified, including 47 sequences from IMGT® and 222 sequences retrieved from 32 genomes (Table 1, Table S1). Among these newly identified TRC sequences, most of which were not previously reported. The number of TRCs in platypus was consistent with that from Parra et al. (47). Recently, Breaux et al. (48) reported the four canonical TCR loci in Florida manatee, where the numbers for each type of TRCs were consistent with ours. All of these newly identified sequences were further classified by phylogenetic trees (Figures S3,S4). Each cluster grouped independently, indicating a clear relationship for each identified gene. We further classified these sequences into three categories: intact genes, partial genes and pseudogenes as described in the methods (Figure 1). The proportion of pseudogenes for *TRAC, TRBC, TRGC*, and *TRDC* were 15.9, 2.4, 3.9, and 2.6%, respectively. We noticed that the fraction of partial genes was higher in some species, such as for pig (*Sus scrofa*) (55%) and giant panda (*Ailuropoda melanoleuca*) (75%) (Figure 1). However, the fractions of partial genes were not correlated with the contig N50 values of the examined genome assemblies (*p* = 0.580), suggesting that the current TRC gene numbers retrieved from the genomes were acceptable for our analysis. As for sequence characteristics, the four types of TRC genes kept conserved Characteristic IMGT® Residues, i.e., 1st-CYS and 2nd-CYS, while some variation caused by mutations occurred at N-glycosylation sites.

For the four types of TRC genes, we only retrieved the first three exons with the exon/intron lengths referenced by human TRCs from IMGT®. *TRAC, TRBC*, and *TRDC* genes showed conserved sequence lengths and Characteristic IMGT® Residues among the examined mammals. For *TRGC* genes, all sequences showed similarities mainly in conserved lengths of EX1 (110 amino acids) and EX3 (47 amino acids) with slight variations caused by indels. However, considerable variations in sequence lengths and amino acid residues were identified throughout connecting regions of *TRGC* genes in human, mouse and ruminants, which were caused by EX2 duplication or triplication. Among these mammals, we found that monotremates, primates, rodents, and carnivores have double, triple or more *TRGC* EX2, while chiroptera and cetaceans have only one *TRGC* EX2. The *TRGC* sequences were longest in platypus, caused by its possessing five copies of EX2, whereas the shortest were from cetaceans, which have contracted EX2 with only 12 amino acid residues (at least 16 amino acid in other mammals).

### TRC Repertoires and Evolution

Gene numbers of the four conventional types of TRCs in the examined mammals were varied. In general, most species possessed single copy of *TRAC* and *TRDC* genes (Figure 1). Two copies of *TRAC* genes were identified in Weddell seals (*Leptonychotes weddellii*), walrus (*Odobenus rosmarus*), pig, minke whale (*Balaenoptera acutorostrata*), bowhead whale (*Balaena mysticetus*), and sperm whale (*Physeter macrocephalus*). For *TRDC*, only giant panda and Egyptian fruit bat possessed 2 sequences. Recurrent duplication and loss of the *TRBC* gene might be occurred during evolution, and the gene numbers varied from 1 to 5. As for the *TRGC* gene, there was a distinct increase of gene numbers in carnivores (9) that were maintained in this order with only slight variations (5–8). The number decreased to 1–4 in hippopotamus and cetaceans.

### Differences in TRC Repertoires in Mammals With Divergent Niches

**Figure 2:**
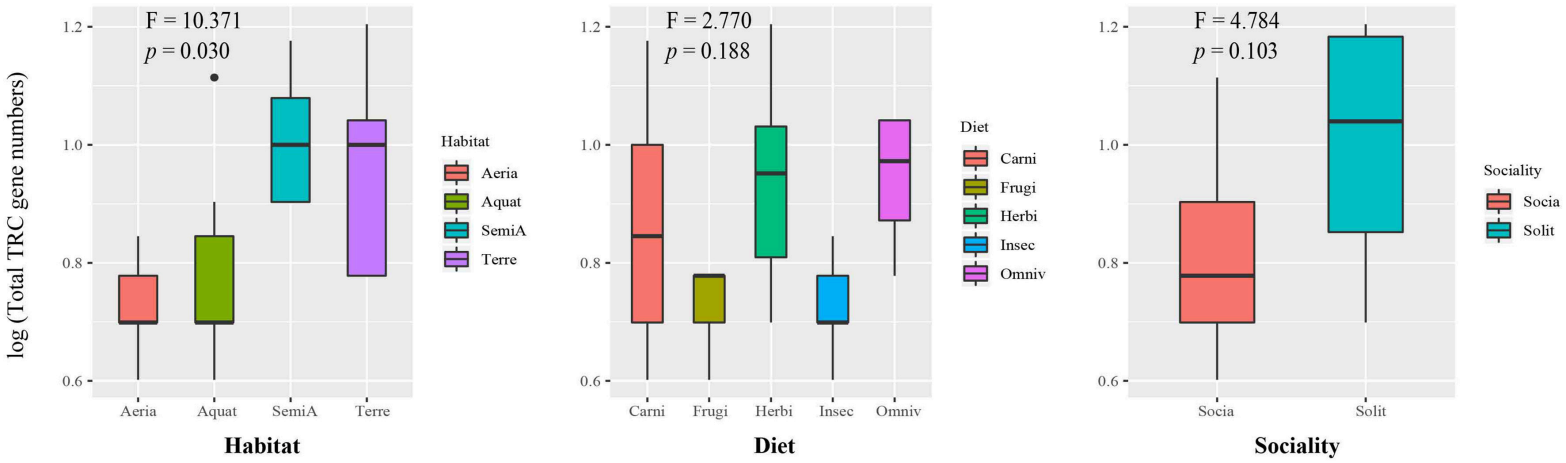
Box plot for comparison of total TRC repertoires among groups of divergent ecological niches. X axis: categories of ecological factors. Y axis: total number of TRC genes after log transformation. *F*-value and *p*-value on the boxplot are extracted from results of phyANOVA. Aquat-Aquatic; SemiA-SemiAquatic; Terre-Terrestrial; Carni-Carnivore; Frugi-Frugivore.

To test the hypothesis that the difference of TRC repertoires may be driven by different ecological factors, we employed a phylogenetic comparative method taking into account the influence of evolutionary relationships between species called phyANOVA (49) to compare the gene number of total TRCs among specified niches groups. There were significant differences in TRC gene numbers among the four types of habitat (*F*-value = 10.371, *p* = 0.030) (Figure 2, Table S4), suggesting that habitat niche is useful for predicting the gene number of TRCs in these mammals. Similar procedures were carried out in each of the four TRC genes. Results showed that the difference among habitat groups was significant for *TRGC* genes (*F*-value = 22.547, *p* = 0.001). In addition, no significant difference was identified among groups based on diet and sociality (*p* > 0.05) (Table S4). Here, we used the total number of TRCs, but the results were essentially the same when pseudogenes were excluded (Table S4).

### Correlation Between Ecological Factors and the Number of TRC Genes

We further performed PGLS regression analyses to investigate which ecological factors affected the TRC number while statically controlling for phylogeny. According to the results, habitat showed the most significant correlation with the number of *TRGC* genes among the three examined predictor variables (*p* < 0.001; Table 2-1). The observation suggest that mammals that inhabit different habitats tend to have different *TRGC* gene repertoires. We then conducted multivariate PGLS regression analyses to access the influence of each ecological factor on the number of TRC genes that is independent from habitat. Surprisingly, the influence that interplay of habitat and sociality on the TRC repertoires was significant (*p* < 0.01) (Table 2-2), indicating that the current TRC repertoires have been shaped by interplay of habitat and sociality. However, diet did not show significant correlations when phylogeny and habitat were statistically controlled (*p* > 0.05). Also, although we used the total TRC repertoires for analyses, the results are essentially the same for the correlation between TRC (*TRGC* genes) and habitat and sociality when removing pseudogenes (Table S5).

**Table 2-1:** Significant results of PGLS regression for TRC gene numbers vs. ecological factors.

**Response variable**	***N***	**Predictor variable**	**Slope**	***p***	***R*^**2**^**	**λ^a^**
*TRC*	37	Habitat	1.125	0.040^*^	0.116	1
*TRGC*	37	Habitat	1.136	< 0.001^***^	0.304	1

**Table 2-2:** Significant results of PGLS multivariate regression for TRC gene numbers vs. ecological factors.

**Response variable**	***N***	**Predictor variable**	**Slope**	***p***	***R*^**2**^**	**λ^a^**
*TRC*	37	Habitat	1.342	0.007^**^	0.281	0.955
		Sociality	3.827	0.012^*^		
*TRGC*	37	Habitat	1.267	< 0.001^***^	0.457	1
		Sociality	2.475	0.004^**^		

a *Pagel's λ for phylogenetic signal (50)*.

* p < 0.05;

** p < 0.01;

*** *p < 0.001*.

In summary, the PGLS analyses demonstrated that habitat and sociality are important factors affecting the number of mammalian TRC genes.

### Divergent Selection on Mammalian TRCs in Niche Adaption

As we have identified the significant differences in TRC gene numbers among ecological factor groups, we next investigated whether diverse selective intensity on mammalian TRC sequences also occurred in response to different niches.

In order to investigate the hypothesis, we first analyzed the dataset using random sites codon models (40) to estimate the overall form and strength of selection acting upon the 37 mammalian TRC sequences. The sites models performed on *TRAC* and *TRDC* genes revealed that the positive model (M8) fitted the data better than the neutral model (M8a). Specifically, the M8 model detected 19 and 14 positively selected sites at *TRAC* and *TRDC* genes, respectively (Table S6). Meanwhile, a total of 23 and 19 sites for *TRAC* and *TRDC* genes were detected by the other three ML models (FEL, REL, and FUBAR). Among which, 9 sites in both *TRAC* and *TRDC* were predicted to be robust sites under positive selection and identified by at least three ML methods.

The branch models was then employed to estimate the specific evolutionary rates of each targeted branches. Free ratio model fitted the data better only for the *TRAC* gene. Generally, the branches with evolutionary rates ω > 1, scattered on the phylogeny (Figure 1). Specifically, we found that there were relatively more positive signals in cetaceans (9/17) and bats (11/27) than that in other groups (artiodactyla: 2/5, carnivora: 2/9, euarchontoglires: 1/5, afrotheria: 1/3).

Further, Clade model C (CmC) was carried out to test if the partitions of habitat (four partitions), diet (five partitions), and sociality (two partitions) (Figure S1) were undergoing divergent selection. All of the partitions for both genes were significantly better fit relative to the M2a_rel model (*p* < 0.01, Table 3), supporting different rates of ω among partitions of habitat, diet and sociality. Notably, among partitions of habitat and sociality, we noticed a completely contrary trend for *TRAC* (habitat: ω_1_ > ω_2_ > ω_4_ > ω_3_, sociality: ω_2_ > ω_1_) and *TRDC* (habitat: ω_3_ > ω_4_ > ω_2_ > ω_1_, sociality: ω_1_ > ω_2_) genes with respect to the evolutionary rates. In addition, we compared the best-fitting CmC model to a null model that the divergent site class of the foreground partition was constrained to equal one. The LRT between these two models is significant (except *TRAC* in diet partitions) (Table S7), suggesting positive selection was the major evolutionary pattern of TRC genes.

**Table 3:** Results of clade model C (CmC) analyses on mammalian TRCs.

**Gene**	**Partition^a^**	**Model**	**np**	**ln*L***	**Parameters^b^**			**LRT**	***df***	***p***
					***ω_*0*_***	***ω_*1*_***	***ω_*2*/_ω_*d*_***			
*TRAC*	n/a	M2a_rel	71	5419.92	0.05 (34.5%)	11 (35.6%)	0.39 (29.9%)			
	Habitat	CmC	75	5378.62	0.12 (41.9%)	1 (39.3%)	3.41 (18.8%)	82.60	4	0
							Aquat 4.50			
							Aeria 2.92			
							SemAq 1.84			
							Terre 2.79			
	Diet	CmC	76	5378.42	0.12 (41.9%)	1 (39.6%)	2.79 (18.4%)	82.99	5	< 0.01
							Carni 3.19			
							Frugi 2.34			
							Herbv 3.77			
							Insec 3.21			
							Omniv 2.07			
	Sociality	CmC	73	5376.82	0.12 (41.8%)	1 (39.3%)	1.83 (18.9%)	86.20	2	0
							Socia 2.87			
							Solit 10.76			
*TRDC*	n/a	M2a_rel	71	5622.82	0.36 (21.1%)	1 (53.1%)	0.04 (25.8%)			
	Habitat	CmC	75	5554.56	0.09 (30.9%)	1 (48.3%)	0.32 (20.8%)	136.52	4	0
							Aquat 0.22			
							Aeria 0.34			
							SemAq 4.50			
							Terre 4.48			
	Diet	CmC	76	5585.73	0.09 (30.7%)	1 (47.6%)	0.43 (21.7%)	74.18	5	< 0.01
							Carni 2.09			
							Frugi 8.14			
							Herbv 2.23			
							Insec 0.53			
							Omniv 3.51			
	Sociality	CmC	73	5595.05	0.08 (29.8%)	1 (44.1%)	4.67 (26.1%)	55.55	2	< 0.01
							Socia 2.21			
							Solit 0.28			

*np, number of parameters; lnL, ln likelihood; df, degrees of freedom; n/a, not applicable; Aquat, Aquatic; SemAq, SemiAquatic; Terre, Terrestrial; Carni, Carnivore; Frugi, Frugivore; Herbv, Herbivore; Insec, Insectivore; Omniv, Omnivore; Socia, Sociality; Solit, Solitary*.

a *Partitions listed are explained in Figure S1*.

b *ω values for each site class (ω_0_–ω_2_) are shown with the proportion of each in parentheses. ω_d_ refers to the divergent site class in the CmC models, which has a separate value for each partition: the first value is for the background, followed by the foreground partition(s)*.

## Discussion

### Two General Mechanisms for Mammalian TRCs in Adaption to Habitat and Sociality

In this study, we retrieved four types of canonical TRC sequences from 32 genomes which represented the minimum level of genes numbers. The numbers might be more accurate with the improvement of assembly quality for the genomes. We further examined TRC gene repertoires in 37 mammals with diverse habitats, diets and socialities. Results of phylogenetic comparative methods (phyANOVA and PGLS) and molecular evolutionary analysis revealed two general mechanisms for the fitness of mammals in adaption to habitat and diet.

#### Trade-Off Between TRGC Numbers and Strength of Positive Selection on TRAC in Adaption to Different Habitats

PhyANOVA analysis suggested that *TRGC* numbers were significantly varied across different habitat groups. The difference was obvious between the high number in semiaquatic groups and aerial and aquatic groups with low number (Figure 2, Table S4). However, the free ratio model revealed a relatively higher proportion of signals with positively selected *TRAC* from cetaceans (9/17) and bats (11/27) than that in other groups (Figure 1). Besides, relatively higher rates for *TRAC* and *TRDC* were also detected in cetaceans and bats than terrestrial and semiaquatic mammals by CmC model (Table 3). Thus, we proposed a trade-off between *TRGC* numbers and strength of positive selection on *TRAC* in adaption to different habitats.

#### Balanced Evolutionary Rates Between TRAC and TRDC in Adaption to the Interplay of Habitat and Sociality

Results of PGLS showed a significant correlation between TRC numbers and the interplay of habitat and sociality, suggesting the evolution of TRC numbers was affected by habitat and sociality. Specially, a completely contrary trend of evolutionary rates for *TRAC* and *TRDC* in habitat and sociality was detected by CmC model. Take habitat as an example, the rates of *TRAC* displayed as ω_aquatic_ > ω_aerial_ > ω_terrestrial_ > ω_semiaquatic_, while the rates of *TRDC* was ω_semiaquatic_ > ω_terrestrial_ > ω_aerial_ > ω_aquatic_. Similarly trend for rates of the two genes in sociality was identified (Table 3). Therefore, balanced evolutionary rates between *TRAC* and *TRDC* genes was speculated to a mechanism in adaption to the interplay of habitat and sociality.

Both of these two mechanisms were important for the fitness of mammals in their specific habitat and sociality. Evidences of habitat transition driving the evolution of immune-related genes have been identified on many innate immunity genes (2, 51). Particularly, most researches on the immune-related genes were carried out to explore the potential mechanism for the immunity adaption of cetaceans during their habitat transition (3, 52). At present, no consistent conclusion about the pathogenic pressure in aquatic habitat revealed by MHC polymorphism (53–55). Generally, viruses are ubiquitous in the sea and they are believed to be the major pathogens of the ocean (56, 57). Epidemics of marine pathogens can spread at extremely fast rates (58). In addition, the view that pathogenic pressure is weaker in aquatic environments than in terrestrial habitats is controversial (53, 55). Recent studies on microbiomes have suggested that the taxonomic composition of microbial communities in marine mammals were distinct from those of terrestrial mammals, while the corresponding diversity was not lower than that of terrestrial mammals (59, 60). Thus, the comparable MHC polymorphism in cetaceans compared to that of terrestrial mammals hinted the potential mechanism for their defensing against marine viruses (55). Meanwhile, TCRs recognize the processed antigens presented by MHC during cellular immunity response. Combined with the highly polymorphism of MHC in cetaceans and the two mechanisms for TRC genes in the present study, both promoted the immunity for the response to viruses faced by cetaceans.

The relationship between sickness and social behavior is intricate (61). Typically, the presence of larger social groups leads to the incidence of more common parasites than found with solitary groups (62). However, there is no specific social behavior that is selectively advantageous for certain pathogens (63). Surprisingly, we observed that relatively more TRCs were identified in solitary groups, which may be attributed to the small number of solitary species (4) included in this study. However, the balanced evolutionary rates between *TRAC* and *TRDC* genes in sociality and solitary mammals provided a clue in understanding the relationship between sociality and evolution of immune-related genes. More importantly, correlations regarding pathogen load of hosts and their social behavior may be more useful to explain the relationship between TRCs and sociality in the future. Moreover, our study did not identify significant correlation between TRC repertoires and diet from PGLS analysis. This is likely due in part to the unbalanced species number among the five diet groups, which might obscure signals of significance. To better understand the correlation between diet and evolution of immune-related genes, future studies would benefit from more comprehensive analysis with more relative genes and species with diverse diets.

### Specific Immunity Adaption for Bats

Bats are the only mammals capable of sustained flight and they are also notorious reservoir hosts of several important emerging viruses (5, 64). There was a view that innate immune systems were the key line of defense in their coexistence with viruses (5). Many pattern recognition receptor genes (TLRs and RLRs) that constitute the first line of defense of organisms were subjected to positive selection in bats, indicating their enhancement of capacity in inflammasome assembly during viral infections (2). Specially, antiviral defenses by constitutively expressed IFN-α and expanded and diversified numerous antiviral loci provided them the ability to coexist with viruses (65, 66). In the present study, we investigated the TRC genes that mainly involved in cellular immunity. Fewer TRCs were identified. Combined with the weak positive signals in chiroptera for IGHC genes, a group of genes involved in humoral immunity (67), the two results provided supporting evidence for the vital position of their innate immunity. However, the two general mechanisms proposed in this study might be also important for their coexistence with viruses via T cells. The different proportion of CD4^+^ and CD8^+^ T cells and the constitutively expressed of IL-17, IL-22, and TGF-β mRNA in bats further underlined the roles mediated by T cells (68). To sum up, the difference in innate and adaptive immune responses between bats and mice and humans may account for special viral infection, which also required more studies like immunoglobulin class-specific antibodies for bats (69).

### γδ T Cell Receptor and Species Immunity Adaption

Wild animals face many pressures, like parasites and bacteria that could affect both their health and fitness (70). T cells play vital roles in defensing against pathogens to keep organisms away from diseases. Among these, αβ T cells make up about 90–95% of the circulating T cell pool in most species, and they are the major lymphocytes involved in cellular immunity (71). However, αβ TCRs could only recognize antigens processed and presented by MHC, so they might be more conserved, in the perspective of evolution, than γδ TCRs, which can recognize more promiscuous ligands directly (72). In addition, although the percentage of γδ T cells is low (~5–10%), they can respond to a variety of disease states, including infectious disease responses, wound healing and tissue homeostasis (72, 73). Specifically, γδ T cells are believed to occupy unique temporal and functional niches in host immune defenses. For example, γδ T cells respond earlier than αβ T cells to infections and emerge after pathogen numbers have started to decline (71). Thus, it seems that αβ TCRs are necessary but regular in immunity reactions, but γδ TCR might serve as special guards to protect the organisms in any case of emergency, especially in the wild.

In this study, we noted a significant correlation between *TRGC* and habitat, which reflected the special roles for *TRGC* in immunity adaption among mammals with different habitats. Although there was no significant correlation between *TRBC* numbers and any of the examined ecological factors, we could not despise the roles provided by αβ TCRs in immunity adaption. In addition, *TRBC* gene numbers were diverse across the examined mammals, and only two pseudogenes were identified, which hinted the necessary demand for immune reaction. Further, there might be different evolutionary mechanisms for genes in TRA and TRB loci from TRG and TRD loci. Further analysis of the variable genes for the four canonical TCR loci among mammals is ongoing and beyond the scope of this paper. Given that immune defense is a complicated process that interacts with many gene families to provide disease defense (74, 75), future studies including more genes (or gene families) will be required to further explore the relationship of species-specific adaptions for various ecological niches via T cell receptors.

## Conclusions

This is the first study on the relationships between mammalian TRC genes and niches adaptions. We have demonstrated the divergent evolution of TRC genes in mammalian niches adaption, suggesting that the current TRC repertoires have been shaped by different ecological factors. Specifically, trade-off between *TRGC* numbers and strength of positive selection on *TRAC* and the balanced evolutionary rates between *TRAC* and *TRDC* genes were speculated as two main mechanisms in adaption to habitat and sociality. The results in our study can guide further exploration of species-specific adaptions via T cell receptors.

## Author Contributions

SX and GY conceived the project and designed the experiments. ZZ, YM, and DS performed the phylogenetic comparative methods and molecular evolution analysis. WG, ZY, and RT helped with analysis and organized the Supplementary Files. ZZ wrote the manuscript. LS, SX, and GY improve the manuscript. All authors read and approved the final manuscript.

### Conflict of Interest Statement

The authors declare that the research was conducted in the absence of any commercial or financial relationships that could be construed as a potential conflict of interest.
