# Supplementary material for: Divergent Evolution of TRC Genes in Mammalian Niche Adaptation
Source: Front Immunol. 2019 Apr 24;10:871. doi: 10.3389/fimmu.2019.00871 (PMC6491686; doi:10.3389/fimmu.2019.00871)
Supplement: Supplementary file 2 [file Data_Sheet_2.docx]

Table S2 Scaffold information for each newly identified genes in this study.

| Loci | Scaffold | Gene name |
| --- | --- | --- |
| TRA | NW_006383530.1 | Lwed_TRAC1 |
|  | NW_006384351.1 | Lwed_TRAC2 |
|  | NW_004450616.1 | Oros_TRAC1p |
|  | NW_004450616.1 | Oros_TRAC2p |
|  | NW_004444204.1 | Tman_TRAC |
|  | NW_007907051.1 | Umar_TRACic |
|  | NW_007370840.1 | Efus_TRAC |
|  | KE749803.1 | Ehel_TRAC1 |
|  | NW_017731735.1 | Harm_TRAC |
|  | NW_005370741.1 | Mbra_TRAC |
|  | NW_006300366.1 | Mdav_TRAC |
|  | NW_005871345.1 | Mluc_TRAC |
|  | KI032460.1 | Mlyr_TRAC1 |
|  | NW_015504389.1 | Mnat_TRAC |
|  | NW_006436757.1 | Pale_TRAC |
|  | KE821390.1 | Ppar_TRAC |
|  | NW_011888994.1 | Pvam_TRAC |
|  | NW_015495256.1 | Raeg_TRAC |
|  | KI126459.1 | Rfer_TRAC |
|  | NW_017739261.1 | Rsin_TRAC |
|  | NKPW01005007.1 | Hamp_TRAC |
|  | NW_006726798.1 | Bacu_TRAC2 |
|  | NW_006733011.1 | Bacu_TRAC1p |
|  | NW_006724793.1 | Bacu_TRAC3 |
|  | NW_017844172.1 | Ttru_TRAC |
|  | NW_004438590.1 | Oorc_TRAC |
|  | NW_006774793.1 | Lvex_TRAC |
|  | NW_006712781.1 | Pcat_TRAC1 |
|  | NW_006715633.1 | Pact_TRAC |
|  | NW_019160932.1 | Dleu_TRAC |
|  | scaffold27 | Neas_TRAC |
|  | scaffold_1763 | Bmys_TRAC2p |
|  | scaffold_3058 | Bmys_TRAC1 |
|  | NC_010449.5 | Sscr_TRAC1 |
|  | NC_010443.5 | Sscr_TRAC2ic |
|  | NW_003218322.1 | Amel_TRACp |
|  | NW_003573524.1 | Lafr_TRAC |
|  | NC_019837.1 | Nleu_TRAC |
|  | PTTO01001689.1 | Oana_TRACic |
| TRB | NW_004444029.1 | Tman_TRBC1 |
|  | NW_004444029.1 | Tman_TRBC2 |
|  | NW_006384713.1 | Lwed_TRBC1 |
|  | NW_006384713.1 | Lwed_TRBC2 |
|  | NW_004451143.1 | Oros_TRBC1 |
|  | NW_004451143.1 | Oros_TRBC2 |
|  | NW_007907159.1 | Umar_TRBC1 |
|  | NW_007907159.1 | Umar_TRBC2 |
|  | NW_007920882.1 | Umar_TRBC3 |
|  | NW_007918157.1 | Umar_TRBC4 |
|  | NW_007370745.1 | Efus_TRBC |
|  | AWHC01247684.1 | Ehel_TRBC |
|  | NW_017732466.1 | Harm_TRBC1 |
|  | NW_017732466.1 | Harm_TRBC2 |
|  | NW_017732466.1 | Harm_TRBC3 |
|  | NW_005356173.1 | Mbra_TRBC1 |
|  | NW_005356173.1 | Mbra_TRBC2 |
|  | NW_005356173.1 | Mbra_TRBC3 |
|  | NW_005295658.1 | Mbra_TRBC4 |
|  | NW_006294067.1 | Mdav_TRBC1 |
|  | NW_006347152.1 | Mdav_TRBC2p |
|  | NW_005871189.1 | Mluc_TRBC1 |
|  | NW_005871189.1 | Mluc_TRBC2 |
|  | KI022523.1 | Mlyr_TRBC |
|  | NW_015504218.1 | Mnat_TRBC1 |
|  | NW_015504218.1 | Mnat_TRBC2 |
|  | NW_006490953.1 | Pale_TRBC3 |
|  | NW_006434839.1 | Pale_TRBC1 |
|  | NW_006434839.1 | Pale_TRBC2 |
|  | KE883724.1 | Ppar_TRBC |
|  | NW_011889110.1 | Pvam_TRBC2 |
|  | NW_011920401.1 | Pvam_TRBC1 |
|  | NW_015494636.1 | Raeg_TRBC1 |
|  | NW_015494636.1 | Raeg_TRBC2 |
|  | KI139385.1 | Rfer_TRBC |
|  | NW_017739569.1 | Rsin_TRBC1 |
|  | NKPW01004888.1 | Hamp_TRBC1ic |
|  | NKPW01008365.1 | Hamp_TRBC2ic |
|  | NKPW01026994.1 | Hamp_TRBC3ic |
|  | NW_006727908.1 | Bacu_TRBC2 |
|  | NW_006727908.1 | Bacu_TRBC4 |
|  | NW_006727908.1 | Bacu_TRBC5 |
|  | NW_006727908.1 | Bacu_TRBC3 |
|  | NW_006725732.1 | Bacu_TRBC1 |
|  | scaffold_2377 | Bmys_TRBC1 |
|  | scaffold_2841 | Bmys_TRBC2 |
|  | NW_017843107.1 | Ttru_TRBC |
|  | NW_004438464.1 | Oorc_TRBC1 |
|  | NW_004439609.1 | Oorc_TRBC2 |
|  | C48679325 | Neas_TRBC1ic |
|  | C47802868 | Neas_TRBC2ic |
|  | scaffold206 | Neas_TRBC3ic |
|  | scaffold206 | Neas_TRBC4ic |
|  | NW_019161543.1 | Dleu_TRBC1 |
|  | NW_019160856.1 | Dleu_TRBC2 |
|  | NW_006793703.1 | Lvex_TRBC |
|  | NW_006722871.1 | Pcat_TRBC1 |
|  | NW_006717431.1 | Pcat_TRBC2 |
|  | NW_006718520.1 | Pcat_TRBC3_ |
|  | NC_010460.4 | Sscr_TRBC1 |
|  | NC_010460.4 | Sscr_TRBC2 |
|  | NC_010460.4 | Sscr_TRBC3 |
|  | NW_003218064.1 | Amel_TRBC1 |
|  | NW_003218064.1 | Amel_TRBC2 |
|  | NW_003227687.1 | Amel_TRBC3 |
|  | NW_003239474.1 | Amel_TRBC4 |
|  | NW_003573503.1 | Lafr_TRBC1 |
|  | NW_003573503.1 | Lafr_TRBC2 |
|  | NC_019828.1 | Nleu_TRBC1 |
|  | NC_019828.1 | Nleu_TRBC2 |
|  | PTTO01002549.1 | Oana_TRBC1 |
|  | PTTO01002549.1 | Oana_TRBC2 |
| TRD | NW_006384351.1 | Lwed_TRDC |
|  | NW_004450416.1 | Oros_TRDC |
|  | NW_004444204.1 | Tman_TRDC |
|  | NW_007925226.1 | Umar_TRDC |
|  | KE748585.1 | Ehel_TRDC |
|  | NW_017731735.1 | Harm_TRDC |
|  | NW_006300366.1 | Mdav_TRDC |
|  | KI032460.1 | Mlyr_TRDC |
|  | NW_015504389.1 | Mnat_TRDC |
|  | KE863726.1 | Ppar_TRDC |
|  | NW_015495256.1 | Raeg_TRDC1ic |
|  | NW_015494647.1 | Raeg_TRDC2ic |
|  | KI126459.1 | Rfer_TRDC |
|  | NW_017739261.1 | Rsin_TRDC |
|  | NKPW01016575.1 | Hamp_TRDC |
|  | NC_010449.5 | Sscr_TRDC |
|  | NW_003218322.1 | Amel_TRDC1p |
|  | NW_003224378.1 | Amel_TRDC2ic |
|  | NW_003573524.1 | Lafr_TRDC |
|  | NC_019837.1 | Nleu_TRDC |
|  | NW_001764819.1 | Oana_TRDC |
|  | NW_006726798.1 | Bacu_TRDC |
|  | scaffold_2435 | Bamy_TRDC |
|  | NW_017844172.1 | Ttru_TRDC |
|  | NW_004438590.1 | Oorc_TRDC |
|  | scaffold27 | Neas_TRDC |
|  | NW_019160932.1 | Dleu_TRDC |
|  | NW_006774793.1 | Lvex_TRDC |
|  | NW_006716024.1 | Pcat_TRDC |
| TRG | NW_006384010.1 | Lwed_TRGC1ic |
|  | NW_006384010.1 | Lwed_TRGC3ic |
|  | NW_006384010.1 | Lwed_TRGC4ic |
|  | NW_006389006.1 | Lwed_TRGC5ic |
|  | NW_006384010.1 | Lwed_TRGC2 |
|  | NW_004451350.1 | Oros_TRGC1 |
|  | NW_004451350.1 | Oros_TRGC2 |
|  | NW_004451350.1 | Oros_TRGC3 |
|  | NW_004451350.1 | Oros_TRGC4ic |
|  | NW_004451400.1 | Oros_TRGC5ic |
|  | NW_004451498.1 | Oros_TRGC6ic |
|  | NW_004452491.1 | Oros_TRGC7 |
|  | NW_004447093.1 | Tman_TRGC1ic |
|  | NKPW01091998.1 | Hamp_TRGC1ic |
|  | NKPW01056299.1 | Hamp_TRGC2ic |
|  | NKPW01004765.1 | Hamp_TRGC3ic |
|  | NC_010451.4 | Sscr_TRGC1ic |
|  | NC_010451.4 | Sscr_TRGC2ic |
|  | NC_010451.4 | Sscr_TRGC3ic |
|  | NC_010451.4 | Sscr_TRGC4ic |
|  | NC_010451.4 | Sscr_TRGC5p |
|  | NW_003217316.1 | Amel_TRGC1ic |
|  | NW_003217316.1 | Amel_TRGC2ic |
|  | NW_003217330.1 | Amel_TRGC3ic |
|  | NW_003217330.1 | Amel_TRGC4ic |
|  | NW_003217330.1 | Amel_TRGC5ic |
|  | NW_003219519.1 | Amel_TRGC6ic |
|  | NW_003219519.1 | Amel_TRGC7ic |
|  | NW_003219519.1 | Amel_TRGC8ic |
|  | NW_003220193.1 | Amel_TRGC9ic |
|  | NW_003573425.1 | Lafr_TRGC1ic |
|  | NW_003573425.1 | Lafr_TRGC2ic |
|  | NC_019832.1 | Nleu_TRGC1 |
|  | NC_019832.1 | Nleu_TRGC2 |
|  | NW_007907215.1 | Umar_TRGC1ic |
|  | NW_007907215.1 | Umar_TRGC2ic |
|  | NW_007907215.1 | Umar_TRGC3ic |
|  | NW_007907215.1 | Umar_TRGC4ic |
|  | NW_007907215.1 | Umar_TRGC5ic |
|  | NW_007907215.1 | Umar_TRGC6ic |
|  | NW_007907215.1 | Umar_TRGC7 |
|  | NW_007907215.1 | Umar_TRGC8 |
|  | NW_007907215.1 | Umar_TRGC9ic |
|  | NW_007370653.1 | Efus_TRGC |
|  | AWHC01196463.1 | Ehel_TRGCic |
|  | NW_017732331.1 | Harm_TRGC |
|  | NW_005354120.1 | Mbra_TRGC |
|  | NW_006286807.1 | Mdav_TRGC |
|  | NW_005871066.1 | Mluc_TRGC |
|  | KI078916.1 | Mlyr_TRGC |
|  | NW_015504275.1 | Mnat_TRGC1 |
|  | NW_006494608.1 | Pale_TRGC |
|  | AWGZ01327453.1 | Ppar_TRGC1ic |
|  | KE907154.1 | Ppar_TRGC2ic |
|  | AWGZ01431896.1 | Ppar_TRGC3ic |
|  | NW_011889083.1 | Pvam_TRGC |
|  | NW_015495111.1 | Raeg_TRGC |
|  | AWHA01258933.1 | Rfer_TRGC1ic |
|  | AWHA01040730.1 | Rfer_TRGC2ic |
|  | NW_017739991.1 | Rsin_TRGC1 |
|  | PTTO01004519.1 | Oana_TRGC1ic |
|  | PTTO01004519.1 | Oana_TRGC2 |
|  | PTTO01004519.1 | Oana_TRGC3 |
|  | PTTO01004519.1 | Oana_TRGC4p |
|  | NW_017843531.1 | Ttru_TRGC |
|  | NW_004438418.1 | Oorc_TRGC |
|  | scaffold373 | Neas_TRGC |
|  | NW_019160902.1 | Dleu_TRGC |
|  | NW_006782612.1 | Lvex_TRGC |
|  | NW_006713894.1 | Pcat_TRGC |
|  | NW_006728902.1 | Bacu_TRGC1 |
|  | NW_006728902.1 | Bacu_TRGC2ic |
|  | NW_006728902.1 | Bacu_TRGC3ic |
|  | NW_006725829.1 | Bacu_TRGC4ic |
|  | scaffold_292 | Bamy_TRGC1 |
|  | scaffold_292 | Bamy_TRGC3ic |
|  | scaffold_292 | Bamy_TRGC2ic |
| TRM | NW_001631199.1 | Oana_TRMC1 |
|  | NW_001646718.1 | Oana_TRMC2p |
|  | NW_001646718.1 | Oana_TRMC3 |
|  | NW_001676381.1 | Oana_TRMC4p |
|  | NW_001694389.1 | Oana_TRMC5 |
|  | NW_001695729.1 | Oana_TRMC6p |
|  | NW_001697014.1 | Oana_TRMC7 |
|  | NW_001709955.1 | Oana_TRMC8 |
|  | NW_001714428.1 | Oana_TRMC9p |
|  | NW_001737883.1 | Oana_TRMC10 |
|  | NW_001741082.1 | Oana_TRMC11p |
|  | NW_001773338.1 | Oana_TRMC12p |
|  | NW_001773338.1 | Oana_TRMC13 |
|  | NW_001789434.1 | Oana_TRMC14 |
|  | NW_001794165.1 | Oana_TRMC15p |
|  | NC_008803.1 | Mdom_TRMC1 |
|  | NC_008803.1 | Mdom_TRMC2 |
|  | NC_008803.1 | Mdom_TRMC3 |
|  | NC_008803.1 | Mdom_TRMC4 |
|  | NC_008803.1 | Mdom_TRMC5 |
|  | NC_008803.1 | Mdom_TRMC6 |
|  | NC_008803.1 | Mdom_TRMC7 |
|  | NC_008803.1 | Mdom_TRMC8 |
|  | NC_008803.1 | Mdom_TRMC9 |
