# Supplementary material for: Divergent Evolution of TRC Genes in Mammalian Niche Adaptation
Source: Front Immunol. 2019 Apr 24;10:871. doi: 10.3389/fimmu.2019.00871 (PMC6491686; doi:10.3389/fimmu.2019.00871)
Supplement: Supplementary file 4 [file Data_Sheet_4.docx]

Table S4 Results of phyANOVA

| Niches | Gene | *F* value | *p* value | *p* value of postdoc | | | |  |  |
| --- | --- | --- | --- | --- | --- | --- | --- | --- | --- |
|  |  |  |  | Aeria vs. Aquat | Aeria vs. SemiA | Aeria vs. Terre | Aquat vs. SemiA | Aquat vs. Terre | SemiA vs. Terre |
| Total TRCs | |  |  |  |  |  |  |  |  |
| Habitat | *TRC* | 10.371 | **0.030** | 0.960 | **0.030** | 0.252 | **0.030** | 0.426 | 0.960 |
|  | *TRGC* | 22.547 | **0.001** | 1.000 | **0.006** | 0.060 | **0.006** | 0.060 | 1.000 |
| TRCs after removing pseudogenes | | | | |  |  |  |  |  |
| Habitat | *TRC* | 10.139 | **0.039** | 0.730 | **0.018** | 0.248 | **0.050** | 0.480 | 0.730 |
|  | *TRGC* | 19.909 | **0.003** | 1.000 | **0.006** | 0.076 | **0.006** | 0.076 | 1.000 |

Abbreviation: Aquat-Aquatic; SemAq-SemiAquatic; Terre-Terrestrial; Socia-Sociality; Solit-Solitary.

The method for postdoc is holm (Holm, 1979).

The significant *p* values (*p*<0.05) are in bold.

Holm. (1979). A simple sequentially rejective multiple test procedure. Scand J Stat. 6, 65–70. doi:10.1007/BF00139637
