# Supplementary material for: Divergent Evolution of TRC Genes in Mammalian Niche Adaptation
Source: Front Immunol. 2019 Apr 24;10:871. doi: 10.3389/fimmu.2019.00871 (PMC6491686; doi:10.3389/fimmu.2019.00871)
Supplement: Supplementary file 5 [file Data_Sheet_5.docx]

Table S5-1 Significant Results of PGLS Regression for TRC Gene Numbers (pseudogenes excluded) versus Ecological Factors

| Response N Predictor Slope *p* R^2^ λ^a^ | | | | | | |
| --- | --- | --- | --- | --- | --- | --- |
| Variable Variable | | | | | | |
| *TRC* | 37 | Sociality | 1.048 | 0.028^*^ | 0.131 | 0.949 |
| *TRGC* | 37 | Habitat | 1.000 | 0.002^**^ | 0.250 | 1 |
| *TRAC* | 37 | Sociality | -0.525 | 0.025^*^ | 0.135 | 0.721 |
| Table S5-2 Significant Results of PGLS Multivariate Regression for TRC Gene Numbers (pseudogenes excluded) versus Ecological Factors | | | | | | |
| Response N Predictor Slope *p* R^2^ λ^a^ | | | | | | |
| Variable Variable | | | | | | |
| *TRC* | 37 | Habitat | 1.220 | 0.005^**^ | 0.281 | 0.892 |
|  |  | Sociality | 3.209 | 0.017^*^ |  |  |
| *TRGC* | 37 | Habitat | 1.128 | <0.001^***^ | 0.404 | 1 |
|  |  | Sociality | 2.406 | 0.006^**^ |  |  |

*^a^* Pagel’s λ for phylogenetic signal (Pagel 1999).

^*^*p*<0.05; ^**^*p*<0.01; ^***^*p*<0.001

Pagel M. (1999). Inferring the historical patterns of biological evolution. *Nature* 401:877–884. doi: 10.1038/44766
