# Supplementary material for: Divergent Evolution of TRC Genes in Mammalian Niche Adaptation
Source: Front Immunol. 2019 Apr 24;10:871. doi: 10.3389/fimmu.2019.00871 (PMC6491686; doi:10.3389/fimmu.2019.00871)
Supplement: Supplementary file 6 [file Data_Sheet_6.docx]

Table S6 PSSs identified by different ML methods

| Gene | PAML^b^ |  |  |  |  | Datamonkey | | |
| --- | --- | --- | --- | --- | --- | --- | --- | --- |
|  | -ln*L* m8a^a^ | -ln*L* m8^a^ | *ω*^c^ | *p*^d^ | PSS^e^ | FUBAR^f^ | FEL^g^ | REL^h^ |
| *TRDC* | 5624.72309 | 5604.99088 | 2.00255 | <0.001 | 41^*^, **80**^*^, **81**^*^, **89**^**^, **95**^*^, 98^*^, 103^**^, 116^**^, **118**^**^, 126^*^, 127^*^, 135^*^, 137^*^, 140^**^ | **5, 64,** 78**, 80, 81, 89,** 91**, 93, 95, 106, 118** | **5**, 49, **64**, 67, **80**, **81**, **89**, **93**, **95**, **106**, 111, 134 | **5**, 31, 41, **64**, 78, **80**, **81**, **89**, 91, **93**, **95**, 98, **106**, 109, **118** |
| *TRAC* | 5418.81953 | 5373.22515 | 2.58927 | <0.001 | 30^*^, 31^*^, 34^*^, **35**^**^, 36^**^, **37**^**^, 38^**^, 40^*^, 55^**^, 67^*^, 68^*^, 71^**^, **73**^**^, 74^**^, **78**^*^, **79**^**^, 84^*^, 85^*^, 86^*^ | **35, 37, 43, 62, 73, 75, 78, 87,106** | 12, **35**, **37**, **43**, 46, **62**, 66, **73**, **75**, **78**, **87**, 89, **106** | 12, **35**, 36, **37**, 38, **43**, 45, 55, **62**, 67, 71, **73**, 74, **75**, 77, **78**, 79, 86, **87**, 89, **106** |

^a^ Two alternative nested models, one “neutral” and the other including one class of sites with *d*_N_/*d*_S_>1, were compared in an LRT

^b^ Codons identifed by more than three ML methods are bolded. Moreover, if the site identifed by all methods are shown in bold and underlined

^c^ *ω*s, estimated *d*_N_/*d*_S_ of the sites under selection in M8

^d^ *p* value for nested models comparison. Signifcance: ^**^*p*<0.01, ^***^*p*< 0.001

^e^ Codons with posterior probabilities> 95% in the BEB analyses, ^*^: *p*>95%; ^**^: *p*> 99%

^f^ Codons with posterior probabilities > 90%

^g^ Codons with *p* values < 0.1

^h^ Codons with Bayes factors >50
