# Supplementary material for: Divergent Evolution of TRC Genes in Mammalian Niche Adaptation
Source: Front Immunol. 2019 Apr 24;10:871. doi: 10.3389/fimmu.2019.00871 (PMC6491686; doi:10.3389/fimmu.2019.00871)
Supplement: Supplementary file 7 [file Data_Sheet_7.docx]

Table S7 Model comparison between CmC (*ω* estimated) and NULL model (*ω* ratio constrained equal to one)

| Gene | Partitions | Model | | | | *p* value |
| --- | --- | --- | --- | --- | --- | --- |
|  |  | *ω*=1 | | CmC (*ω* estimated) | |  |
|  |  | np | ln*L* | np | ln*L* |  |
| *TRAC* | Diet | 75 | 5378.429 | 76 | 5378.422 | 0.903 |
|  | Habitat | 74 | 5380.58 | 75 | 5378.617 | 0.048 |
|  | Sociality | 72 | 5382.082 | 73 | 5376.815 | 0.001 |
| *TRDC* | Diet | 75 | 5589.373 | 76 | 5585.727 | 0.007 |
|  | Habitat | 74 | 5573.394 | 75 | 5554.557 | <0.001 |
|  | Sociality | 72 | 5601.905 | 73 | 5595.046 | 0.000 |
