# Supplementary material for: Divergent Evolution of TRC Genes in Mammalian Niche Adaptation
Source: Front Immunol. 2019 Apr 24;10:871. doi: 10.3389/fimmu.2019.00871 (PMC6491686; doi:10.3389/fimmu.2019.00871)
Supplement: Supplementary file 8 [file Image_1.pdf]

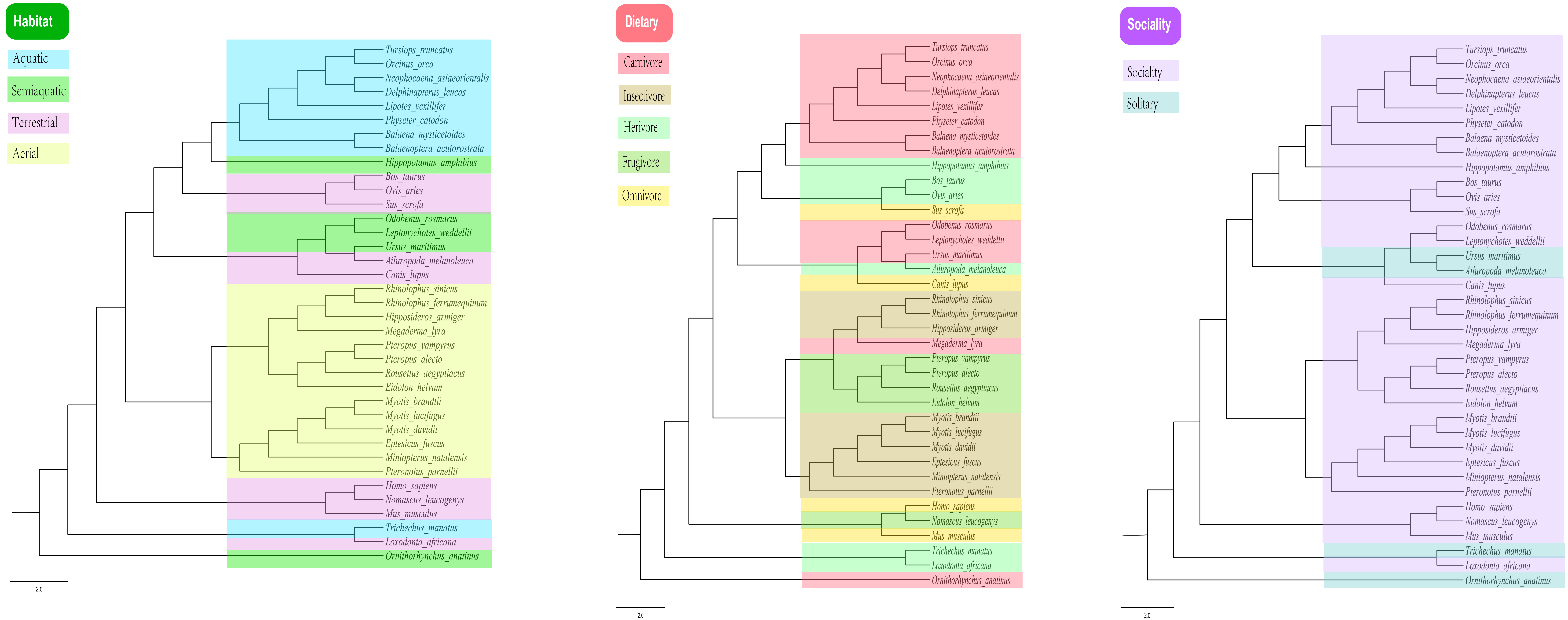

Figure S1 Phylogenetic tree of 37 species with category of ecological niches. Reference for the niches was listed in Table S2. The phylogenetic tree was modified from a widely accepted phylogeny of mammals (Ranwez et al., 2007; Teeling et al., 2005; Zhou et al., 2011).
