# Supplementary material for: Divergent Evolution of TRC Genes in Mammalian Niche Adaptation
Source: Front Immunol. 2019 Apr 24;10:871. doi: 10.3389/fimmu.2019.00871 (PMC6491686; doi:10.3389/fimmu.2019.00871)
Supplement: Supplementary file 9 [file Image_2.pdf]

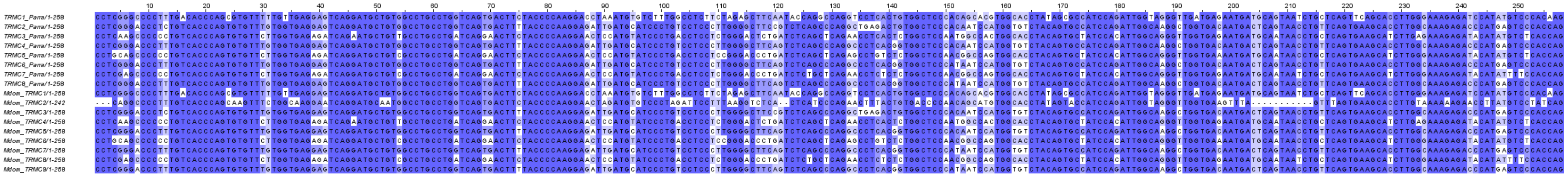

Figure S2-A. Sequences alignment for *TRMC*s in opossum. The alignment was extracted from Jawview (Waterhouse et al., 2009). The sequences were colored by %identity in Jawview. The eight *TRMC* sequences marked with *TRMC*_parra were extracted from Parra et al., 2008 according to the position given in the additional file 1. The last nine sequences marked with Mdom_TRMC were the newly identified *TRMC* sequences in this study. The numbers follow each sequence name were sequence length.

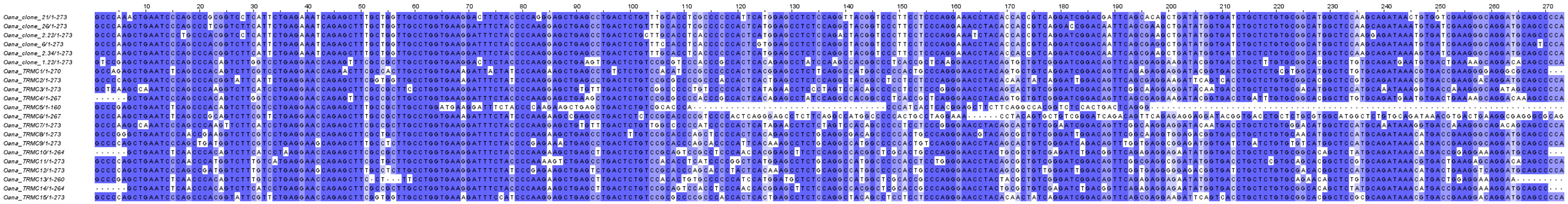

Figure S2-B. Sequences alignment for *TRMC*s in platypus. The alignment was extracted from Jawview (Waterhouse et al., 2009). The sequences were colored by %identity in Jawview. The first six sequences marked with Oana_clone were from Wang et al., 2011. The following 15 sequences marked with Oana_*TRMC* were the newly identified *TRMC* sequences in this study. The numbers follow each sequence name were sequence length.

Waterhouse AM, Procter JB, Martin DMA, Clamp M, Barton GJ. "Jalview Version 2 - a multiple sequence alignment editor and analysis workbench".Bioinformatics (2009) 25 (9) 1189-1191. doi: 10.1093/bioinformatics/btp033

Parra ZE, Baker ML, Hathaway J, Lopez AM, Trujillo J, Sharp A, et al. Comparative genomic analysis and evolution of the T cell receptor loci in the opossum Monodelphis domestica. BMC Genomics. (2008) 9:111. doi: 10.1186/1471-2164-9-111
