# Supplementary material for: Divergent Evolution of TRC Genes in Mammalian Niche Adaptation
Source: Front Immunol. 2019 Apr 24;10:871. doi: 10.3389/fimmu.2019.00871 (PMC6491686; doi:10.3389/fimmu.2019.00871)
Supplement: Supplementary file 10 [file Image_3.pdf]

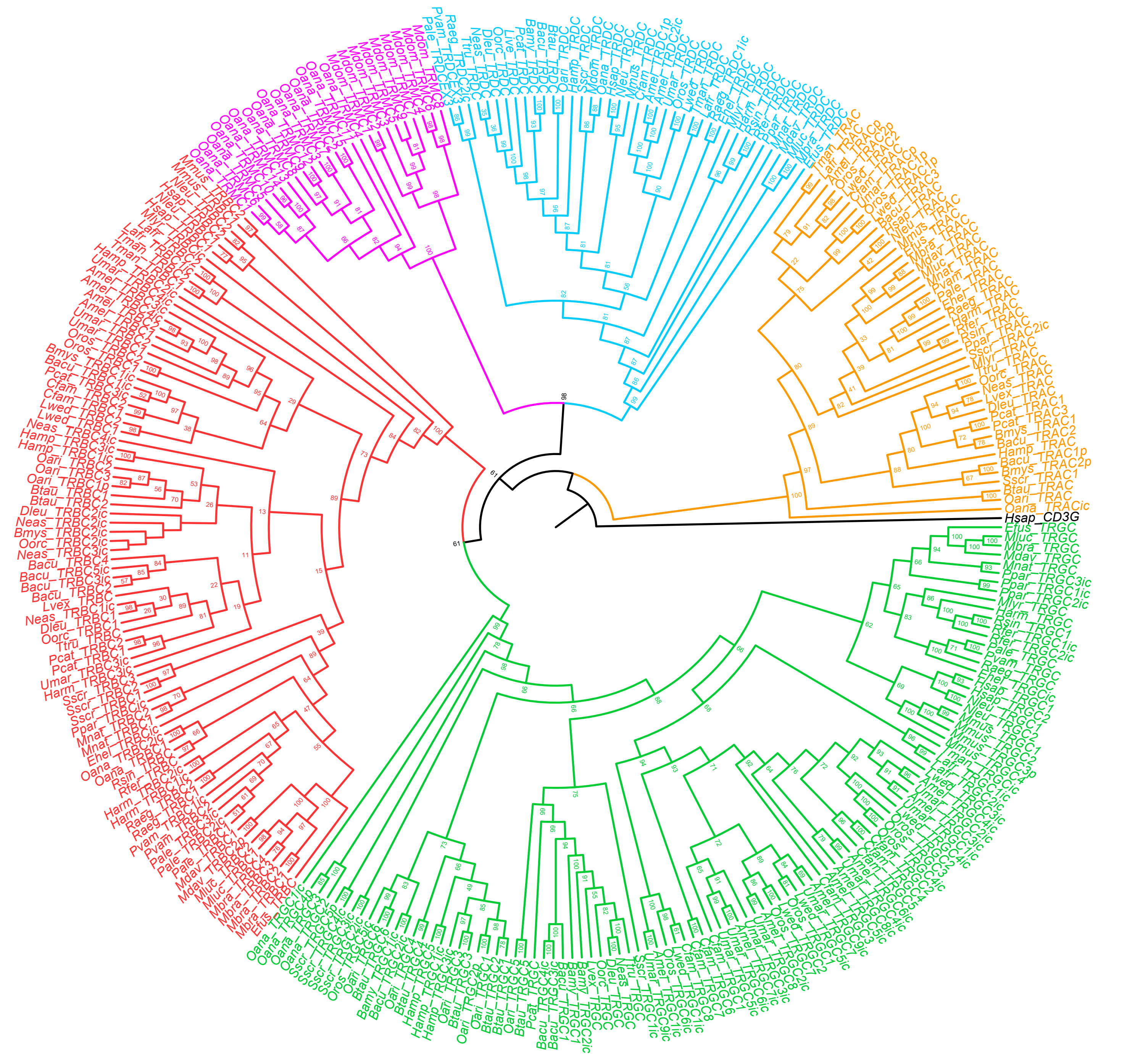

Figure S3 Phylogenetic tree of mammalian TRCs reconstructed by IQ-TREE. Each cluster with different colors indicates corresponding types of TRC genes. Yellow: *TRAC*. Red: *TRBC*. Blue: *TRDC*. Green: *TRGC*. Purple: TRMC. Outgroup: *Hsap_CD3G* (NCBI accesion number: XM_006718941.3)
