# Supplementary material for: Divergent Evolution of TRC Genes in Mammalian Niche Adaptation
Source: Front Immunol. 2019 Apr 24;10:871. doi: 10.3389/fimmu.2019.00871 (PMC6491686; doi:10.3389/fimmu.2019.00871)
Supplement: Supplementary file 11 [file Image_4.pdf]

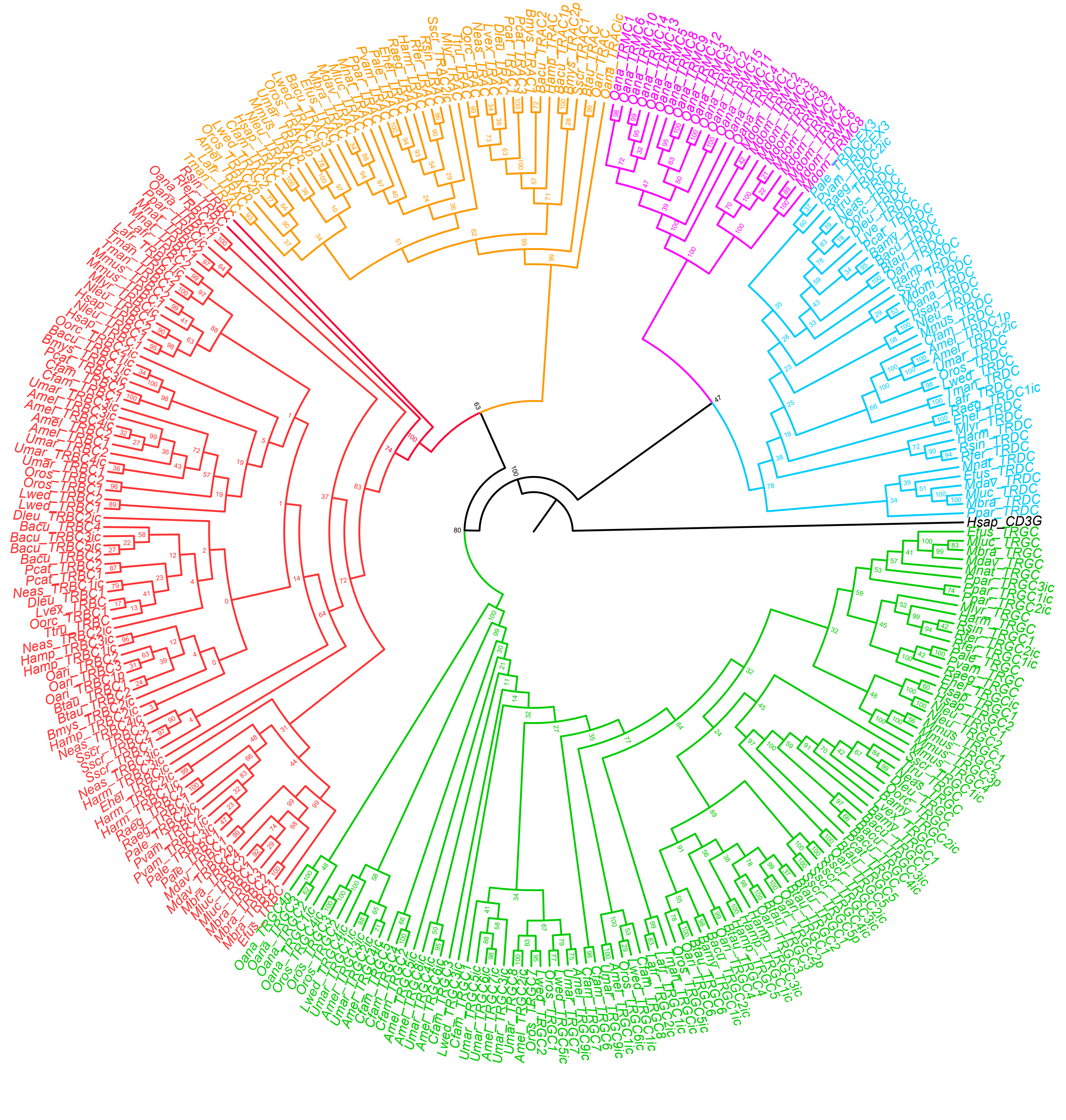

Figure S4 Phylogenetic tree of mammalian TRCs reconstructed by Mrbayes. Each cluster with different colors indicates corresponding types of TRC genes. Yellow: *TRAC*. Red: *TRBC*. Blue: *TRDC*. Green: *TRGC*. Purple: TRMC. Outgroup: *Hsap_CD3G* (NCBI accesion number: XM_006718941.3)
